# Platelet Rich Plasma and Hyaluronic Acid Blend for the Treatment of Osteoarthritis: Rheological and Biological Evaluation

**DOI:** 10.1371/journal.pone.0157048

**Published:** 2016-06-16

**Authors:** Fabrizio Russo, Matteo D’Este, Gianluca Vadalà, Caterina Cattani, Rocco Papalia, Mauro Alini, Vincenzo Denaro

**Affiliations:** 1 Department of Orthopaedic and Trauma Surgery, Campus Bio-Medico University of Rome, Via Alvaro del Portillo 200, 00128, Rome, Italy; 2 AO Research Institute Davos, Clavadelerstrasse 8, 7270, Davos, Switzerland; National University of Ireland, Galway (NUI Galway), IRELAND

## Abstract

**Introduction:**

Osteoarthritis (OA) is the most common musculoskeletal disease. Current treatments for OA are mainly symptomatic and inadequate since none results in restoration of fully functional cartilage. Hyaluronic Acid (HA) intra-articular injections are widely accepted for the treatment of pain associated to OA. The goal of HA viscosupplementation is to reduce pain and improve viscoelasticity of synovial fluid. Platelet-rich plasma (PRP) has been also employed to treat OA to possibly induce cartilage regeneration. The combination of HA and PRP could supply many advantages for tissue repair. Indeed, it conjugates HA viscosupplementation with PRP regenerative properties. The aim of this study was to evaluate the rheological and biological properties of different HA compositions in combination with PRP in order to identify (i) the viscoelastic features of the HA-PRP blends, (ii) their biological effect on osteoarthritic chondrocytes and (iii) HA formulations suitable for use in combination with PRP.

**Materials and Methods:**

HA/PRP blends have been obtained mixing human PRP and three different HA at different concentrations: 1) Sinovial, 0.8% (SN); 2) Sinovial Forte 1.6% (SF); 3) Sinovial HL 3.2% (HL); 4) Hyalubrix 1.5% (HX). Combinations of phosphate buffered saline (PBS) and the four HA types were used as control. Rheological measurements were performed on an Anton PaarMCR-302 rheometer. Amplitude sweep, frequency sweep and rotational measurements were performed and viscoelastic properties were evaluated. The rheological data were validated performing the tests in presence of Bovine Serum Albumin (BSA) up to ultra-physiological concentration (7%). Primary osteoarthritic chondrocytes were cultured *in vitro* with the HA and PRP blends in the culture medium for one week. Cell viability, proliferation and glycosaminoglycan (GAG) content were assessed.

**Results:**

PRP addition to HA leads to a decrease of viscoelastic shear moduli and increase of the crossover point, due to a pure dilution effect. For viscosupplements with HA concentration below 1% the viscoelasticity is mostly lost. Results were validated also in presence of proteins, which in synovial fluid are more abundant than HA.

Chondrocytes proliferated overtime in all different culture conditions. The proliferation rate was higher in chondrocytes cultured in the media containing PRP compared to the cultures with different HA alone. GAG content was significantly higher in chondrocytes cultured in PRP and HL blend.

**Discussion:**

We investigated the rheological and biological properties of four different HA concentrations when combined with PRP giving insights on viscoelastic and biological properties of a promising approach for future OA therapy. Our data demonstrate that PRP addition is not detrimental to the viscosupplementation effect of HA. Viscosupplements containing low HA concentration are not indicated for combination with PRP, as the viscoelastic properties are lost. Although having the same rheological behavior of SF and HX, HL was superior in stimulating extracellular matrix production *in vitro*.

## Introduction

Osteoarthritis (OA) is the most common musculoskeletal disease, affecting approximately 27 million adults in the United States [1] and more than 39 million in Europe [2]. It represents an important socioeconomic burden due to its high prevalence and associated progressive disability and pain. OA is a chronic degenerative joint disease characterized by imbalanced homeostasis and decreased extracellular matrix (ECM) deposit, thinning and eventual wearing of articular cartilage (AC) as the result of prolonged mechanical and inflammatory stress [3]. The AC has a limited healing potential due to its vascularity and the low mitotic activity of chondrocytes. It may result in the involvement of other joint tissues with sclerosis, edema of the subchondral bone and inflammation of the synovium leading to stiffness, swelling and restricted joint motion negatively affecting patient mobility and quality of life [4].

The goals for the treatment of OA are relieving pain, improving function and quality of life. Current conservative management is mainly symptomatic and consists of physiotherapy, analgesics, non-steroid and steroid anti-inflammatory drugs and corticosteroid injections [5]. However, they provide only temporary and limited benefit and some have potential safety concerns. Moreover, added to the short-term action they do not impact on the natural history or progression of the disease [6] nor have clinically relevant chondro-protective effects [7]. Joint replacement surgery represents the definitive option for severe OA in patients who are not responsive to conservative therapy [4]. Although being an effective procedure, surgery is associated with some serious complications such as infection, deep-vein thrombosis, and prosthesis loosening without considering the limited lifespan.

Therefore, innovative methods are needed to more effectively treat OA, directly addressing the disease process. Among conservative treatments, viscosupplementation with hyaluronic acid (HA), whose natural form is present in healthy joint fluid, has been extensively used as an adjunct in cartilage repair and its use is widespread in clinical practice [8–11]. HA is a non-sulfated glycosaminoglycan (GAG) in cartilaginous ECM maintaining chondrocyte functions [12] and a component of synovial fluid responsible for its viscoelasticy [8]. *In vitro* studies showed that it modulates inflammation inhibiting matrix metalloproteinases (MMPs) [13].

In OA the lower concentration and molecular weight of HA reduce the viscoelasticity of the synovial fluid and increase the friction between articular surfaces [14]. Therefore, HA intra-articular injections are widely accepted for the treatment of pain associated to OA [15, 16]. The goal of viscosupplementation is reducing pain and improving viscoelasticity of synovial fluid [17], thus reducing the disability and improving the function of the joint [18–20]. However, even though HA viscosupplementation has a symptomatic effect, it does not act on the degenerative process of the AC [21] and its long-term effects remain unclear.

Over the past decade, biological research has highlighted the importance of growth factors in musculoskeletal diseases and increasing attention has been paid to the development and use of platelet rich plasma (PRP) [22, 23]. PRP is defined as an autologous blood product obtained by centrifuged-spun whole blood [24] that has a concentration of platelets above baseline levels [25, 26]. It contains a highly concentrated amount of growth factors, which are released through degranulation of platelets. It has been widely used in sports medicine and orthopedics [27–31] and its beneficial effects on cartilage regeneration have been well clarified [32, 33]. It has been shown that PRP could increase ECM synthesis, inhibit inflammation and enhance chondrogenesis in AC [34]. Moreover, the clinical efficacy of PRP in the treatment of knee OA has been tested. Kon et al. have shown a statistically significant improvement of clinical scores in 100 patients affected by knee OA and treated with 3 intra-articular PRP injections [23].

The combination of HA and PRP could supply many advantages for tissue repair [35–37]. Indeed, the HA viscosupplementation, at different concentrations and molecular weights, may reduce pain without, however, promoting repair or anabolic processes in cartilage. At the same time, PRP may reduce pain enhancing the anabolic activity of chondrocytes and production of ECM.

The aim of this study was to evaluate rheological and biological properties of different HA compositions in combination with PRP in order to identify (i) the viscoelastic features of HA-PRP blends, (ii) their biological effect on osteoarthritic chondrocytes and (iii) HA formulations suitable for use in combination with PRP.

## Materials and Methods

### Platelet Rich Plasma Production

The ethics committee of Campus Bio-Medico University of Rome approved the study, and all participants provided written informed consent prior to enrollment. The blood samples from 10 consenting blood donors were used for the experiments. Eight milliliters of peripheral blood was collected in one RegenLab THT tubes® and processed according to the manufacturer instructions. Briefly, the tube was centrifuged at 3100 rpm for 9 minutes. Through centrifugation, the gel contained in the tube separated red blood cells from PRP. After shaking, the PRP was aspirated with an 18-gauge needle and stored. Platelet concentrates were pooled to obtain about 80 mL in plasma suspension with platelets count of 1.8 x10^6^ cells/mL and residual white blood cells (WBC) 0.9 x 10^6^ /mL. Platelets and WBC count were determined with an automated haematology system according to the pre-analytic recommendations. Total amount of PRP pool was then fractionated in small aliquots before freezing at -20°C, under controlled temperature.

### Hydrogel assembly

Mixing and polymerization experiments have been performed using human PRP and four different HA concentrations: 1) Sinovial, 0.8% (SN), 16mg/2ml (Molecular Weight—MW: 800–1200 kDa; IBSA) 2) Sinovial Forte 1.6% (SF), 32mg/2ml (MW: 800–1200 kDa; IBSA); 3) Sinovial HL 3.2% (HL), 64mg/2ml (MW: High 1100–1400 kDa, Low 80–100 kDa; IBSA); 4) Hyalubrix 1.5% (HX), 30mg/2ml (MW: 1500–2000 kDa; Fidia Farmaceutici). PRP/HA blends (PRP/HA) have been obtained mixing the components in volumetric ratio 1:1.

For the rheological tests, 1:1 v:v combinations of phosphate buffered saline (PBS) and the four HA types were used as control. Bovine Serum Albumin (BSA)-containing HA/PRP samples were prepared pre-mixing BSA powder with HA for a total final concentration of 3.5% and 7%.

### Rheological measurements

Rheological measurements were performed on an Anton PaarMCR-302 stress controlled rheometer equipped with a cone-plate geometry of 50 mm diameter and 1 degree angle and Peltier temperature control device with thermostatic hood. Gap was set at 100 μm, correspondent to the cone truncation. An amplitude sweep at the angular frequency of 10 rad/s was performed before every oscillatory measurement in order to set amplitude within the linear viscoelastic range. A 2% strain resulted suitable for all samples and was adopted for each oscillatory experiment. Frequency sweep was performed between 0.1 and 100 rad/s; temperature was set at 37.00 ± 0.02°C. Hydrogels where prepared directly on the bottom element of the rheometer, carefully mixing the components as previously described. A layer of low-viscosity silicon oil was distributed along the interface meniscus to avoid evaporation. A waiting time of 10 minutes was set before frequency sweep measurement in order to allow complete equilibration. Rotational measurements were performed with the same geometry and at physiological temperature. Viscosity was measured as a function of the shear rate for dγ/dt between 10^−1^ and 10^2^ s^-1^. Experiments were run in triplicates.

### Cell isolation and culture

Human chondrocytes were collected aseptically from three OA patients (aged 66, 73 and 75 years) who had undergone knee joint replacement and were provided informed consent, as required by Ethics Committee of Campus Bio-Medico University Hospital. The chondrocytes were isolated according to a standardized procedure [38]. Briefly, cartilage was minced and digested at 37°C with pronase solution (5% Fetal Bovine Serum—FBS, 1% penicillin/streptomycin—P/S, 0.2% pronase; Calbiochem) for 90 minutes and with collagenase solution (5% FBS, 1%P/S, 0.02% collagenase II; Sigma-Aldrich) for 18 hours in Dulbecco's Modified Eagle Medium—DMEM (Sigma-Aldrich). For routine cultures, chondrocytes were maintained in DMEM (Sigma-Aldrich) with 10% FBS (Gibco) and 1% P/S (Lonza) in humidified atmosphere containing 5% CO_2_. Cells were maintained in culture to reach 70% confluence and used for the experiments at passage 1.

### Cell Viability and Proliferation assay

Chondrocytes were seeded into 96-well plate at a density of 5x10^4^ cells/ml in DMEM with 10% FBS and 1% P/S at 37°C for 24h, then the medium was removed from each well and replaced with growing media supplemented with 20% of PRP, SF/PRP, HL/PRP, HX/PRP. SN/PRP blend was not biologically evaluated due to its rheological properties, which make it unsuitable for the proposed use. HA was used at a concentration of 2 mg/ml. To create in vitro OA model, the pro-inflammatory cytokine interleukin-1β (IL-1β) was applied in the cultures at a concentration of 5 ng/ml as previously described [39]. Cells cultured in growing media alone were used as a control. Cell viability and proliferation were analyzed at different time points (1, 3 and 7 days) after incubation with 100 μl of CellTiter-Glo Luminescent Cell Viability Assay (Promega) at 37°C for 3h. The viable cells were measured quantitatively using an optical density plate reader (490 nm; Tecan infinite M200 PRO).

### Glycosaminoglycan content

Chondrocytes were seeded into 24-well plate at a density of 5x10^5^ cells/ml in DMEM with 10% FBS and 1% P/S at 37°C for 24h, then the medium was removed from each well and replaced with growing media supplemented with 20% of PRP, SF/PRP, HL/PRP, HX/PRP as described above. GAG content normalized to DNA was assessed at 7 days of culture. Cell cultures were washed with PBS and digested with 100 μl of papain solution (0.25mg/ml in 50 mM Phosphate buffer, pH 6.5 containing 5mM cysteine–hydrochloride and 5mM ethylenediaminetetraacetic acid—EDTA) overnight at 65°C. GAGs were measured by reaction with 1,9-dimethylmethylene blue (DMMB, Polysciences) using chondroitin sulfate (Sigma-Aldrich) as standard. Measurements of absorption were performed at 530 nm (Tecan infinite M200 PRO).

DNA content was assessed using PicoGreen® Assay (Sigma) as per manufacturer’s guidelines on the cells extracts. A standard curve based on known concentration of DNA was used to determine the DNA content. The sample fluorescence was measured using a microplate reader (Tecan infinite M200 PRO) at 460 nm and 540 nm respectively.

Data are expressed as percentage of GAG normalized to DNA content of chondrocytes cells cultured in growing media for 7 days.

### Statistical analysis

For cell proliferation and total GAG/DNA of *in vitro* studies, chondrocytes from three different donors were used, and each experiment was performed in triplicate samples. SPSS 19.0 statistical software was used for statistical analysis (significance at p < 0.05). One-way analysis of variance (ANOVA) with Tukey post hoc test was used to determine differences among the different groups. A p-value <0.05 was considered statistically significant.

## Results

### Rheological evaluation

**Fig 1:**
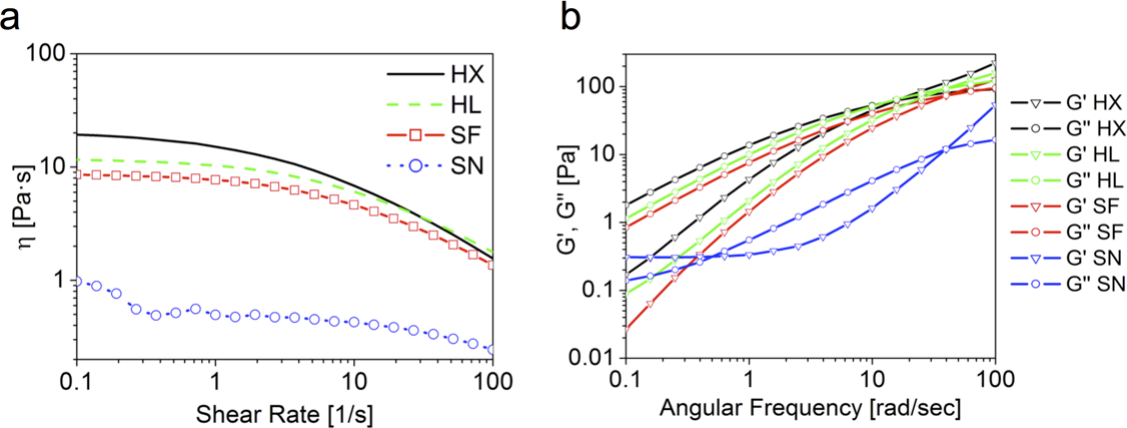
**(A)** Viscosity curves of the pure viscosupplements. The graph represents the viscosity as a function of the shear rate. For all specimens the typical shear-thinning behavior of HA is observed. SN displays viscosity one order of magnitude lower than the other specimens. **(B)** Mechanical spectra of the pure viscosupplements. The graph represents the shear storage modulus G' (triangles) and the shear loss modulus G" (circle) as a function of the angular frequency.

A range of different non-crosslinked hyaluronans commonly used for viscosupplementation in OA was selected for this study. Fig 1 illustrates the rheological characteristics of these products. The viscosity curves (Fig 1A) showed for all the typical non-Newtonian behavior of hyaluronan in aqueous solution. HX had a viscosity of 15.1 Pa·s, HA64 10.3 Pa·s, and SF 7.7 Pa·s at the shear rate of 1 s^-1^. SN, owing to the lower HA concentration, displayed a much lower viscosity of 0.5 Pa·s. The trend described was mirrored by the frequency sweep analysis (Fig 1B), with the shear moduli values in the same sequence of the viscosities. For all specimens the typical HA profile was found, with shear loss modulus G" prevalence at lower frequency and a crossover point after which the shear storage modulus G' was higher. The only outlier was again SN for which at physiologically relevant frequencies (1–10 rad/s) shear moduli were below 1 Pa.

**Fig 2:**
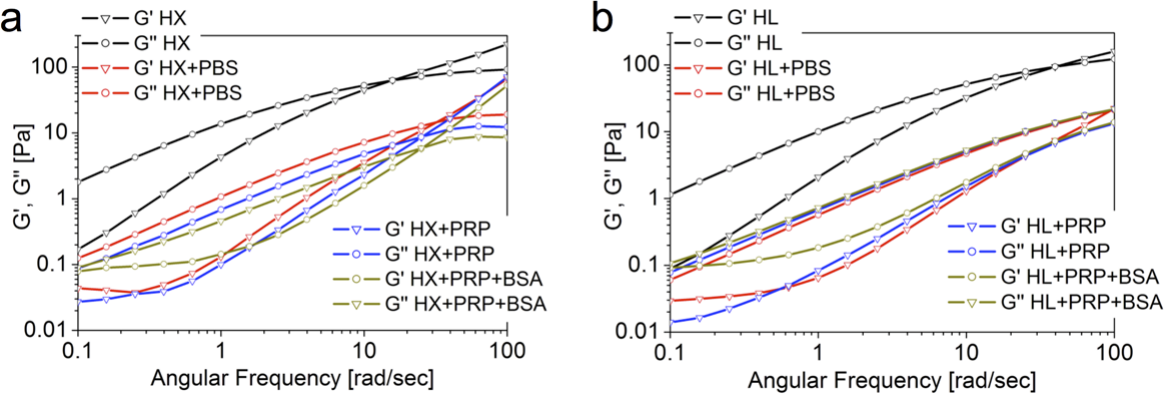
**(A):** Comparison of the mechanical spectrum of pure HX and its combinations: pure HX (black); HX+PBS 1:1 (red); HX+PRP 1:1 (blue); HX+PRP pre-added to 7% BSA (dark yellow). All HX combinations show a similar rheological profile, which is dictated by the dilution effect **(B):** Comparison of the mechanical spectrum of pure HL and its combinations: pure HL (black); HL+PBS 1:1 (red); HL+PRP 1:1 (blue); HL+PRP pre-added to 7% BSA (dark yellow). All HL combinations show a similar rheological profile, which is dictated by the dilution effect.

Fig 2A compares the frequency sweep of pure HX and HX mixed with PRP. With the PRP addition two effects were visible: 1) muduli decreased more than one order of magnitude and 2) the crossover point increased from 10 to 25 rad/s. For comparison, the same analysis was performed for a mixture of HX + PBS. The obtained curves were retracing the profile of HX + PRP. This observation was confirmed by the viscosity curves of the same specimens (not shown). The same measurement performed in presence of BSA up to 7% did not reveal any perturbation from the profile seen, indicating that albumin does not disrupt the interactions or create further entanglements altering the rheological profile of HA-PRP mixtures. HL (Fig 2B) and SF displayed the same trend as HX upon mixing with PRP or PBS.

**Fig 3:**
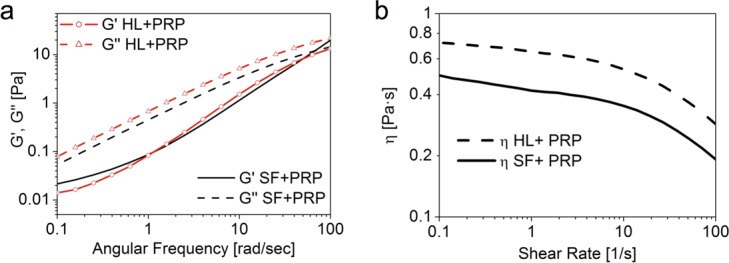
**(A):** Mechanical spectra of HL and SF in combination with PRP. **(B):** Viscosity curve of HL and SF in combination with PRP.

In spite of the double total HA concentration, HL and SF showed a very similar rheological profile (Fig 1). After PRP addition (Fig 3A) shear moduli decreased of about one order of magnitude, but the similarity between HL and SF was maintained. Viscosity curves (Fig 3B) gave similar behaviour, with drop in absolute values but HL always over SF.

### Biological evaluation

**Fig 4:**
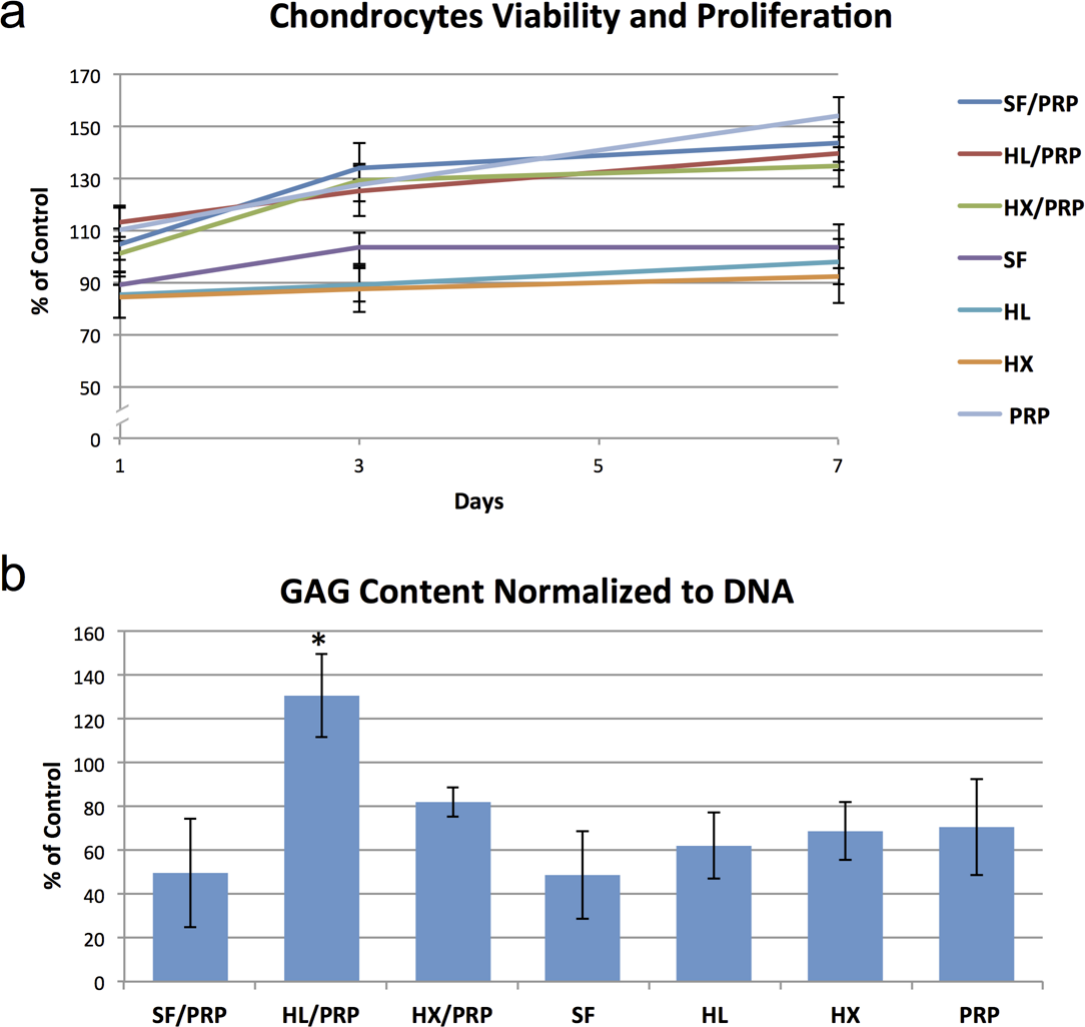
**(A):** Cell viability and proliferation overtime of chondrocytes cultured under different conditions normalized to the rates of chondrocytes cultured in growing media at day 0, 3 and 7. **(B):** GAG content normalized to DNA of chondrocytes cultured for 7 days under different conditions compared to the GAG content of chondrocytes cultured in growing media (*: p<0.05).

Cell viability and proliferation was measured at each time point and was expressed as the percentage of each cell culture normalized to chondrocytes cultured in standard growing media. Chondrocytes proliferated overtime in all culture conditions. The proliferation rate was higher in chondrocytes cultured in the media containing PRP compared to the cultures with different HA alone (Fig 4A).

The amount of sulphated GAG was evaluated using DMMB assay and is presented in terms of GAG per DNA as percentage of the control (chondrocytes cultured in standard growing media). Chondrocytes cultured in HL/PRP blend showed a significant higher GAG production compared to other culture conditions (Fig 4B).

## Discussion

The use of HA in combination with PRP is rapidly expanding in the clinical practice [35, 36]. However, the influence of PRP on the rheological and biological properties of HA-based viscosupplements is still mostly unknown. Therefore, the goal of this study was to investigate the rheological and biological behavior of different HA-based clinically available viscosupplements when combined with PRP.

HA has been shown to be an anti-inflammatory molecule able to increase the viscosity of synovial fluid and promote endogenous production of HA [19, 40]. However, HA is a polysaccharide and its intra-articular administration can only have a limited biological effect on cartilage repair. Therefore, it comes natural that HA viscosupplementation may require additional components for improved bioactivity. PRP has gained great importance in the treatment of OA due to its simple production, low cost and minimally invasive nature. It has been shown that it can play a crucial role in tissue repair. Indeed, *in vivo* studies have demonstrated that it improves histologic appearance of the articular cartilage repaired tissue [41, 42], increasing its stiffness [41, 43] and proteoglycan [44] and type II collagen content [45]. Indeed, growth factors in PRP are active cellular signals, which can modify gene expression of OA chondrocytes. However, PRP do not contain macromolecules such as HA, which is a key ingredient for triggering the regeneration of connective tissues [46, 47] and fundamental for the viscosupplementation needed for OA treatment. Developing a new formulation of PRP and HA may benefit from their dissimilar biological mechanisms, combining the rheological properties of HA with the regenerative potential of PRP, thus, leading to a novel and more effective treatment for OA. Indeed, HA and PRP were reported to slow down cartilage degeneration recovering chondrocytes and restoring ECM production, thus, proving this new treatment strategy as a valid intra-articular regenerative and anti-inflammatory therapy for OA [48].

The viscosupplements selected for this study embrace a wide range of HA concentrations and molecular weights and their rheological properties vary accordingly. The dynamic viscosity ranges from 15.1 to 0.5 Pa·s, representing most of the injectable HAs for viscosupplementation clinically available. For all shear thinning behavior was displayed, i.e. decreasing viscosity for increasing shear rate due to polymeric chains disentanglement and progressive alignment along the shear direction.

PRP is constituted by cellular material, polymeric components, proteins, phospholipids and other substances. Therefore its presence might influence the rheological profile perturbing the interaction among the HA chains. As shown in Fig 2, the addition of PRP to the HA viscosupplements leads to a decrease of about one order of magnitude of the viscoelastic shear moduli. In order to evaluate if these changes were due to cellular debris presence, chemical interaction between the PRP components and HA or a simple dilution effect, the same measurement was performed mixing HA with PBS at the same ratio of the PRP dilution. The equivalence of the profile obtained indicated that the changes in the rheological profile after PRP addition can be attributed to a pure dilution effect, without active disruption of interactions between the HA chains. This is an indication that PRP addition is not detrimental to the viscosupplementation effect of HA.

Albumin and globulin are the major components of synovial fluid, where total protein content is even higher than HA [49]. Therefore, the study of the rheological behavior of injectables for viscosupplementation needs to take into account the effect of protein presence [50–52]. Based on our results, the viscoelastic profile of our HA solutions with or without PRP is not modified by albumin presence. The same holds true also for supraphysiological concentrations up to 7%. This non-sensitivity to albumin presence is a further validation of the suitability of PRP additions to HA viscosupplement solutions.

Despite the double concentration of HL compared to SF, their viscoelastic profile was very similar. HL is featured by a bimodal molecular weight profile distribution, with a high and low molecular weight fractions combined. SF is featured by the same high molecular weight fraction of HL but is devoid of the low molecular weight fraction. Therefore, the similar rheological profile for HL and SF could be attributed to the fact that the oscillatory rheological behavior is dominated by the fraction with higher molecular weight, which is the same for both formulations. Despite the double concentration, within HL the extra content of HA is of much lower molecular weight, and therefore not able to significantly perturb the viscoelasticity profile imposed by the longer chains. On the other side, HL displays systematically higher viscosity after mixing with PRP within the whole shear rate region explored (Fig 3B). By comparison with literature data [53], we can observe that HX, HL, SF and their mixture with PRP display viscoelastic properties falling within the range of healthy synovial fluid.

The viscosupplement with HA concentration below 1% (SN) showed a significantly lower viscosity and viscoelastic shear moduli compared to HX, HL and SF. As a consequence, after addition of PRP or PBS its viscoelasticity dropped to reach values considerably lower than the usual range of healthy synovial fluid or viscosupplements [53]. Therefore, viscosuplements with low HA concentration are deemed as less appropriate for combination with PRP for clinical use in OA.

We also explored the biological effects of HA and PRP used in conjugation on relevant cells *in vitro*. Chondrocytes have been cultured in presence of IL-1β in order to mimic degenerative conditions of OA [39]. Cell viability and proliferation rate increased in all culture conditions over time. However, PRP with or without HA significantly boosts cell proliferation and viability. On the other hand, the anabolic properties of OA chondrocytes, measured as GAG synthesis, were significantly increased when HL was used in combination with PRP. This stimulation effect was attributed to the presence of the low molecular weight fraction.

Chen. *et al*., investigated the therapeutic effects of HA and PRP on OA.[48] Using an *in vitro* OA chondrocyte model, they demonstrated that the combination of HA (low MW: 50–120 kDa) and PRP induce chondrogenesis via regenerative signaling associated to an inhibition of the inflammation pathways. These effects were synergistic compared to either HA or PRP alone. Moreover, the study showed that HA and PRP effectively recovered OA symptoms in an OA animal model (anterior cruciate ligament transection model). In agreement with our study, Chen e*t al*. showed how a low MW HA and PRP can significantly enhance the anabolic activity of OA chondrocytes. Therefore we may hypothesize that the presence of a Low MW HA is beneficial for the achievement of a synergistic effect between the PRP and HA. Based on our results, using a medium or high MW HA devoid of the low MW component results in similar rhelogical properties but the boost effect in terms of GAG synthesis is not observed.

However, in a clinical view, translating basic knowledge from bench to bedside is still challenging. As potential novel therapeutic strategies are being developed, some limits of OA research still persist [54]. Indeed the conclusions are limited by the only *in vitro* evaluation performed on monolayer chondrocytes cultures. Further investigations are necessary to verify if this effect is also observed in animal models. Moreover, for clinical efficacy verification, three armed randomized studies would be necessary to provide data on the synergistic effect between low MW HA, high MW HA and PRP on OA management. It would guide more appropriate and timely therapeutic interventions, which could allow more efficient interference with disease progression.

## Conclusion

We investigated the rheological and biological properties of four different HA concentrations when combined with PRP. Our data demonstrate that PRP addition is not detrimental to the viscosupplementation effect of HA. Formulations with HA concentration below 1% display significant drop of viscoelastic properties upon mixing with PRP. Although having the similar rheological behavior of SF and HX, HL in combination with PRP displays superior capacity of stimulating ECM production *in vitro*, possibly due to the presence of a low MW HA fraction. This study presents useful insights on the viscoelastic and biological properties of HA/PRP combinations as promising approach for OA therapy.
